# Rubella Immunity among Italian Female Healthcare Workers: A Serological Study

**DOI:** 10.3390/ijerph17217992

**Published:** 2020-10-30

**Authors:** Luca Coppeta, Cristiana Ferrari, Ilaria Iannuzzi, Iacopo D’Alessandro, Ottavia Balbi, Antonio Pietroiusti, Marco Trabucco Aurilio

**Affiliations:** 1 Department of Biomedicine and Prevention, University of Rome Tor Vergata, 00133 Rome, Italy; pietroiu@uniroma2.it; 2 School of Occupational Medicine, University of Rome Tor Vergata, 00133 Rome, Italy; cristiana.ferrari@ptvonline.it (C.F.); ilaria.iannuzzi3@hotmail.it (I.I.); iacopodalessandro@libero.it (I.D.); ottaventidue@hotmail.it (O.B.); 3 Department of Medicine and Health Sciences, University of Molise, 86100 Campobasso, Italy

**Keywords:** occupational transmission of rubella, rubella, vaccination strategy

## Abstract

Rubella, also known as German measles or three-day measles, is an infectious disease caused by virus of the genus Rubivirus, which may be prevented by vaccination. The infection is potentially dangerous for non immune subjects, although 20–50% of infected subjects are asymptomatic. Healthcare workers (HCWs) have an increased potential exposure to rubella in comparison to the general population, putting them and their patients at risk of infection and its complications. In 2019, 20 cases of rubella have been reported in Italy. According to the Italian National Immunization and Prevention Plan, HCWs should provide a written certification of vaccination for rubella or serological evidence of protective antibodies. The aim of the study was to evaluate the rubella immunization status in female HCWs of the teaching hospital Policlinic Rome Tor Vergata (PTV) of childbearing age. For this purpose, we retrospectively checked the serologic values of rubella-specific IgG antibodies analyzing the clinical records of the HCWs of undergoing the occupational health surveillance program from January 1st to June1st 2020. Five hundred fourteen HCWs with a mean age of 23.19 (range 19–37, DS: 2.80) were included: 90.3% (464) showed a protective antibody titre. The mean value of the anti-rubella IgG was 49.59 IU/mL. Our study shows a non-protective anti rubella IgG titre in a substantial percentage of HCWs (9.7%). As vaccine protection decreases over the years and the risk of congenital rubella syndrome (CRS) in vaccinated subjects should not be underestimated, we suggest routine screening of the immunological status followed by the administration of a third dose of vaccine if the antibody titre becomes non-protective.

## 1. Introduction

Rubella is a vaccine-preventable disease caused by virus of the genus Rubivirus. Also known as German measles or three-day measles, it is characterized by a rash that begins on the face and then spreads to the rest of the body. Although 20–50% of infected subjects are asymptomatic the infection is potentially dangerous for non-immune subjects. Typical signs and symptoms are low-grade fever, headache, general discomfort, cough, pink eye, enlarged lymph nodes, muscle pain, runny or stuffy nose [1].

Infection acquired during pregnancy can cause the congenital rubella syndrome (CRS) with a clinical spectrum spanning from birth defects, to abortion or premature birth, to deafness [2,3]. Worldwide, it is estimated that almost 100,000 children with CRS were born each year [4].

The stage of pregnancy at which infection occurs, is a major determinant of the fetal involvement; non immune subjects infected during the first trimester can transmit the infection to the fetus in 80–100% of cases [5,6,7,8]. The diagnostic criteria for CRS released by the World Health Organization (WHO) include sensorineural deafness, cardiopathy and cataract.

WHO reported approximately 44,896 cases of rubella in 2019, whereas 10,976 cases were reported in 53 European countries in 2018, despite the availability of an effective vaccine in most of countries. In Italy, the median age of the 20 cases occurring in 2019 in seven regions, was 22 years, a typical childbearing age [9,10].

In 2012 WHO launched the “Measles & Rubella Initiative”, a strategic plan covering the time period 2012–2020, including a series of global goals to achieve measles and rubella elimination and to prevent the congenital rubella complications, in at least five WHO regions, by the end of 2020 [11]. The WHO and the Center For Disease Control (CDC) report that although on a worldwide basis three out of ten children are not protected from rubella, the virus has been eradicated in 80 countries, not including Italy, however [12,13,14].

In Italy the rate of rubella infection decreased over last years. Since 2005, there have been two spikes, one in 2008 (30 cases, incidence 5.2 per 100,000 children) and the second in 2012 (21 cases, incidence 3.9 per 100,000 children). Since 2013, the incidence of congenital rubella has been less than 1 case/100,000 children. In 2018, the national vaccination coverage rate for rubella in people aging 18 years (cohort 2000) was 88.4% for the first dose and 82.2% for the second one [9]. Probably due to vaccine hesitancy among population and to the antivaccination movements the rate of vaccinated young populations decreased from 2013 up to 2016, raising again starting from 2017, when compulsory vaccination was introduced. [10].

Healthcare workers (HCWs) have an increased risk of infection due to their occupational exposure, in comparison to the general population [15,16]. According to Italian National Plan for Immunization and Prevention, HCWs should achieve presumptive evidence of immunity to rubella or serological evidence of protective antibodies. For this reason, occupational medicine ambulatories, according to the Italian laws, should promote the measles-mumps-rubella vaccine (MMR). In previous studies, suboptimal immunization rates for measles and mumps were found among HCWs, as shown by the decrease of protective antibody titre over years [17,18,19,20]. MMR is highly effective in preventing rubella, in fact 1-dose vaccine is effective in 95% of the cases and 2-dose vaccine effectiveness is 99% [21]. Even if two doses of MMR vaccine are considered to provide long-lasting immunity, reinfection may nevertheless occur in vaccinated individuals with low levels of antibodies. The frequency and the clinical consequences of this phenomenon are currently unknown, but its occurrence is believed rare. Indeed, although levels of vaccine-induced rubella antibodies might decrease over time, data from surveillance of rubella and CRS suggest that waning immunity with increased susceptibility to rubella disease does not occur. Among persons having had the administration of two doses, approximately 91–100% had detectable antibodies 12 to 15 years later [22].

Vaccination failure, both primary and secondary, is due to maternal antibodies inhibiting an effective immune response to vaccination; incomplete vaccine timeline; reduction of antibody titre, after a very long time, various causes [23]. Rubella linked to the loss of immunity protection is generally mild, however, in women of childbearing age there is the potential risk of congenital rubella syndrome (CRS) [24]. Aim of the study is to evaluate the rubella immunization status in female HCWs, to estimate the reinfection risk and to assess if a third dose should be recommended in order to prevent the congenital rubella syndrome (CRS), according to the Advisory Committee on Immunization Practices (ACIP).

## 2. Methods

This was a retrospective observational study, approved by the Ethical Committee for Research in 89 Human Subjects of the teaching hospital Policlinic Rome Tor Vergata (PTV) (R. S. 193/2018). All procedures performed in the studies were in accordance with the ethical standards of the Institutional Research Committee (approved with authorization number 170) and with the 1964 Helsinki Declaration and its later amendments or comparable ethical standards. We analyzed the clinical records of female HCWs of PTV of childbearing age who underwent the annual occupational medical visit, from 1 January to 1 June 2020. Using ModuLab, the software adopted by the Chemical Analytical Laboratory of PTV, we reported all values of rubella-specific IgG antibodies in a Microsoft Excel worksheet. For each patient, the following data were recorded: age, vaccine doses, age at vaccination, and IgG rubella antibody titre. Rubella-specific IgG antibodies values higher than 10.0 IU/mL were considered protective against disease according to the literature [25]. In PTV, the evaluation of the immunization against rubella is performed by means of the LIAISON^®^ Rubella IgG assay using chemiluminescence immunoassay (CLIA) technology: in this way a semi-quantitative evaluation of specific IgG antibodies to rubella virus in human serum or plasma samples is obtained. 590 subjects were potentially eligible for the study. Subjects with incomplete clinical (36 individuals) or serological (40 subjects) data were excluded from the study, so 514 HCWs were included. We calculated the rate of protected HCWs and the mean value of rubella IgG specific antibodies, in relation to the main variables collected. Statistical analysis was performed by means of SPSS analytic software. We used a chi squared test for dichotomous variables and logistic regression model for multivariate analysis. Having a protective rubella-specific IgG antibodies titre was the dependent variable of a binary logistic regression model, with age, number of doses and time years elapsed from vaccination date as independent variables. T *p* values < 0.05 were considered statistically significant.

## 3. Results

We evaluated the clinical records of 590 female HCWs, undergoing the occupational health surveillance program at Rome Policlinic Tor Vergata. We excluded from the study 76 subjects having uncomplete vaccination data; finally, 514 HCWs were included in the study. Median age of the population was 23.9 years (range: 19–37, DS: 2.80). Median time elapsed from the administration of the last vaccine dose was 16.16 years (DS: 5.28). Main population characteristics are shown in Table 1.

We found 464 HCWs (90.3% of the whole sample) had a protective rubella IgG antibodies value, (95% C.I.: 86.0–93.6%). Immune HCWs among subjects aging <23 years were 90.7% (95% C.I.: 85.1–94.7%) whereas the rate of immunity among HCWs aging 23 years or more was 89.6% (95% C.I.: 81.7–94.9%); *p* = n.s. The rate of protected HCWs was higher among those who received the last dose earlier than 16 years before (97.1–95% C.I.: 84.7–99.9%- vs. 88.0–95% C.I.: 75.7–95.5%; *p* < 0.05) and in HCWs vaccinated with two doses in comparison to those who received one dose (93.2–95% C.I.: 88.9–96.2% vs. 78–95% C.I.: 64.0–88.5%; *p* < 0.05). Average antibody titre was 37.61 IU/mL in HCWs receiving just 1 dose, and 52.50 IU/mL in HCWs receiving 2 doses. Main results are shown in Table 2. n.s. = Not Significant.

We performed a logistic regression type analysis to present odd ratios differences among the different groups considered, based on age and number of doses. We found that vaccination with two doses of MMR was significantly related with serological protection (OR: 3.88; 95% CI: 2.10–7.17; *p* < 0.05) even after controlling for age. The protection rate was higher among HCWs vaccinated with two doses in comparison to those who received 1 dose of MMR and among subjects receiving vaccination less than 15 years before the evaluation (OR: 2.41; 95% C.I.: 1.08–5.33; *p* < 0005) whereas age was not significantly related to immune status (OR: 1.01; 95% C.I.: 0.47–2.13: *p* = n.s.).

## 4. Discussion

The study is focused on the immunological status against rubella in HCWs of a large teaching hospital in Rome. We found a high rate of immune subjects, especially in the HCWs who had received two vaccine doses, even if the protective antibody titre decreases over the years since the last dose. In individuals of general population vaccinated with two doses of MPR, the evidence of non- protective rubella antibody titre isn’t a recommendation for the administration of an additional dose of vaccine, since the protection from clinically significant manifestations of rubella seems to be guaranteed, due to the persistence of the immunological memory. However for HCWs, with a high risk of exposure such as those working in departments of infectious disease or in emergency departments a third dose should be considered, in the case they are women of childbearing age with a low antibody titre, in order to prevent congenital rubella syndrome (CRS) and all the diseases rubella linked [24,26,27].

In Italy, the number of cases of rubella notified in 2019 and the results of our study suggest a risk of virus infection for HCWs, so the Occupational Medicine Service of PTV decided to offer free MMR vaccinations to primary non-immune HCWs and to the ones who, despite two doses, still showed low titre according to the Centers for Disease Control and Prevention’s guidelines, MMR vaccine or MMR serological test is recommended for HCWs that not report this vaccination in anamnesis [24]. Although the administration of a third dose is not supported by the actual recommendations, our study support the need for the immunological screening, and the administration of supplementary doses in order to elicitate and maintain a protective antibody titre among HCWs. Moreover, workplace vaccination seems to be to be cost- effective among these subjects [28], because it is often difficult to find a complete immunization schedules or to trace vaccination data, as there isn’t a centralized registry. In our opinion, an important public health policy to prevent rubella and its complications is based on sensitization of HCWs, especially targeting female HCWs of childbearing age, to get vaccinated, as many individuals remain unprotected due to the vaccine hesitancy.

We also observed that the antibody titre was not related with the age at which the vaccine was administered, in fact the rate of serological protection was the same both in those who had received the vaccination in early childhood (1–3 years old) and in adolescence a finding reported also for HBV vaccinated subjects [29]. The study had some potential limitation: we did not consider the different exposure risk, as the study population is made of HCWs who have a homogeneous risk, and the type of vaccine, since in most cases it was the same.

## 5. Conclusions

Our study shows non-protective anti-rubella IgG titres in a substantial percentage of HCWs (9.7%). As vaccine protection decreases over the years and the risk of congenital rubella syndrome (CRS) in vaccinated subjects should not be underestimated, immunological status should be screened and a third dose of vaccine should be considered if the antibody titre becomes non-protective, particularly in areas of hospitals at high risk for infection.

## Figures and Tables

**Table 1:** Main population characteristics.

Characteristics	N (%)	Mean Age (SD)	Mean Titre (SD)
Total number	514 (100)	23.19 ± 2.80	-
Age	-	-	-
<23	322 (62.6)	-	-
>23	192 (37.4)	-	-
Number of doses received		-	-
1 dose	100 (19.5)	-	37.61
		-	IU/mL ± 55.56
2 doses	414 (80.5)	-	52.50
		-	IU/mL ± 55.71
Time from vaccination		-	-
<16 years	68 (40.5)	-	-
≥16 years	100 (59.5)	-	-

**Table 2:** Main results.

Variables	Total	Percent	Titer > 10.0 IU/mL	Percent	*p*-Value		
			Number				
Years							
				90.7 (95% C.I.: 85.1–94.7)			
<23	322	62.6	292				
					n.s.		
				89.6 (95% C.I.: 81.7–94.9)			
			
≥23	192	37.4	172				
Vaccination							
				78 (95% C.I.: 64.0–88.5)			
1 dose	100	19.5	78				
					<0.01		
				
				93.2 (95% C.I.: 88.9–96.2)			
			
2 doses	414	80.5	386				
Time from							
vaccination							
				97,1 (95% C.I.: 84.7–99.9)			
<16 years	68	40.5	66				
					<0.05		
				88,0 (95% C.I.: 75.7–95.5)			
			
≥16 years	100	59.5	88				

## Key Points

Assessment of rubella is important in healthcare workers because they bear a high risk of infection. Adequate vaccination programs for healthcare workers are necessary.

## References

[1] Centers for Disease Control and Prevention (CDC) Rubella (German Measles, Three-Days Measles); Sign and Symptoms. https://www.cdc.gov/rubella/index.html.

[2] Maldonado Y.A., Long S.S., Pickering L.K., Prober C.G. (2008). Rubella Virus. Principles and Practice of Pediatric Infectious Diseases.

[3] Shukla S., Maraqa N.F. (2020). Congenital Rubella.

[4] George S., Viswanathan R., Sapkal G.N. (2019). Molecular aspects of the teratogenesis of rubella virus. Biol. Res..

[5] Cradock-Watson J.E., Ridehalgh M.K.S., Anderson M.J., Pattison J.R., Kangro H.O. (1980). Fetal infection resulting from maternal rubella after the first trimester of pregnancy. J. Hyg..

[6] Miller E., Cradock-Watson J.E., Pollock T.M. (1982). Consequences of confirmed maternal rubella at successive stages of pregnancy. Lancet.

[7] Giambi C., Del Manso M., Bella A., Filia A., Rota M.C. Rosolia Congenita e in Gravidanza News. http://www.epicentro.iss.it/problemi/rosolia/bollettino.asp.

[8] Jankovic D. (2012). Rubella and CRS overview for WHO Europe. Progress Toward Rubella Elimination and CRS Prevention in Europe.

[9] Filia A., Bella A., Del Manso M., Baggieri M., Marchi A., Bucci P., Magurano F., Nicoletti L., Rota M.C. Morbillo & Rosolia News, N. 56. https://www.epicentro.iss.it/morbillo/bollettino/RM_News_2019_56.pdf.

[10] Filia A., Bella A., Del Manso M., Baggieri M., Marchi A., Bucci P., Magurano F., Nicoletti L., Rota M.C. Morbillo & Rosolia News. https://www.epicentro.iss.it/morbillo/bollettino/RM_News_2019_58.pdf.

[11] Centers for Disease Control and Prevention (CDC) (2013). Rubella and congenital rubella syndrome control and elimination—global progress, 2012. Wkly. Epidemiol. Rec..

[12] Centers for Disease Control and Prevention (CDC) (2005). Progress toward elimination of measles and prevention of congenital rubella infection—European region, 1990–2004. MMWR Morb. Mortal. Wkly. Rep..

[13] Centers for Disease Control and Prevention (CDC) (2013). Prevention of Measles, Rubella, Congenital Rubella Syndrome, and Mumps, 2013: Summary Recommendations of the Advisory Committee on Immunization Practices (ACIP). MMWR.

[14] Grant G.B., Desai S., Dumolard L., Kretsinger K., Reef S.E. (2019). Progress Toward Rubella and Congenital Rubella Syndrome Control and Elimination—Worldwide, 2000–2018. MMWR Morb. Mortal. Wkly. Rep..

[15] Filia A., Bella A., Manso M., Rota C. Istituto Superior di Sanità. Morbillo. Aspetti Epidemiologici. http://www.epicentro.iss.it/problemi/morbillo/epidItalia.asp.

[16] Coppeta L., Biondi G., Perrone S., Pietroiusti A. (2020). Susceptibility to measles among healthcare workers: A cross-sectional serological study. Infect. Dis..

[17] Coppeta L., Pietroiusti A., Morucci L., Neri A., Ferraro M., Magrini A. (2019). Workplace vaccination against measles in a teaching hospital of Rome. J. Hosp. Infect..

[18] Coppeta L., Biondi G., Lieto P., Pietroiusti A. (2019). Evaluation of Immunity to Measles in a Cohort of Medical Students in Rome, Italy. Vaccines.

[19] Coppeta L., Somma G., di Giampaolo L., Bizzarro G., Ippoliti L., Borelli F., Balbi O., Perrone S., Pietroiusti A. (2020). Persistence of antibodies for measles among vaccinated medical students in Italy. Infect. Dis..

[20] Coppeta L., Pietroiusti A., Lieto P., Ferraro M., Grelli S., Stillo M., Magrini A. (2019). Measles immunity in an Italian teaching hospital. Occup. Med..

[21] Bankamp B., Hickman C., Icenogle J.P., Rota P.A. (2019). Successes and challenges for preventing measles, mumps and rubella by vaccination. Curr. Opin. Virol..

[22] Crooke S.N., Haralambieva I.H., Grill D.E., Ovsyannikova I.G., Kennedy R.B., Poland G.A. (2019). Seroprevalence and durability of rubella virus antibodies in a highly immunized population. Vaccine.

[23] Wiedermann U., Garner-Spitzer E., Wagner A. (2016). Primary vaccine failure to routine vaccines: Why and what to do?. Hum. Vaccin. Immunother..

[24] Centers for Disease Control and Prevention (CDC) Immunization of Health-Care Personnel Recommendations of the Advisory Committee on Immunization Practices (ACIP) Recommendations and Reports/Vol. 60/No. 7. https://www.cdc.gov/mmwr/pdf/rr/rr6007.pdf.

[25] Laurence P., Skendzel M.D. (1996). Rubella Immunity Defining the Level of Protective Antibody. Am. J. Clin. Pathol..

[26] Terracciano E., Amadori F., Pettinicchio V., Zaratti L., Franco E. (2020). Strategies for elimination of rubella in pregnancy and of congenital rubella syndrome in high and upper-middle income countries. J. Prev. Med. Hyg..

[27] Marchi S., Viviani S., Montomoli E., Trombetta C.M. (2020). Elimination of congenital rubella: A seroprevalence study of pregnant women and women of childbearing age in Italy. Hum. Vaccin. Immunother..

[28] Coppeta L., Balbi O., Baldi S., Pietroiusti A., Magrini A. (2019). Pre-vaccination IgG screening for mumps is the most cost-effectiveness immunization strategy among Health Care Workers. Hum. Vaccin. Immunother..

[29] Coppeta L., Pompei A., Balbi O., Zordo L.M., Mormone F., Policardo S., Lieto P., Pietroiusti A., Magrini A. (2019). Persistence of Immunity for Hepatitis B Virus among Healthcare Workers and Italian Medical Students 20 Years after Vaccination. Int. J. Environ. Res. Public Health.

